# Suicide myths and preparedness to help among staff at home care and long-term care facilities in Sweden: a quantitative questionnaire-based study

**DOI:** 10.1186/s12877-026-07393-y

**Published:** 2026-04-02

**Authors:** Sabrina Doering, Elias Asteberg, TuvaLisa Berglund, Sara Blom, Stefan Wiktorsson, Sylvie Lapierre, Lena Johansson, Katarina Wilhelmson, Anne Ingeborg Berg, Margda Waern

**Affiliations:** 1 Department of Psychiatry and Neurochemistry, Institute of Neuroscience and Physiology, Sahlgrenska Academy, University of Gothenburg, Blå Stråket 15, SU/Sahlgrenska, Gothenburg, 413 45 Sweden; 2 Department of Psychotic Disorders, Sahlgrenska University Hospital, Region Västra Götaland, Gothenburg, Sweden; 3 Department of Psychology, University of Quebec in Trois-Rivieres, Trois- Rivieres, Canada; 4 Institute of Health and Care Sciences, Sahlgrenska Academy, University of Gothenburg, Gothenburg, Sweden; 5 Department of Addiction Disorders, Sahlgrenska University Hospital, Gothenburg, Region Västra Götaland Sweden; 6 Department of Acute Medicine and Geriatrics, Sahlgrenska University Hospital, Gothenburg, Region Västra Götaland Sweden; 7 Department of Psychology, University of Gothenburg, Gothenburg, Sweden

**Keywords:** Older adults, Suicide, Home care, Long-term care facilities, Suicide myths, Mental disorders, Ageism

## Abstract

**Background:**

Suicide rates are highest among adults aged 65 and older. Given that many older adults receive home care (HC) or reside in long-term care facilities (LTCFs), staff in these settings play a crucial role in suicide prevention. This study assessed the prevalence of suicide myth endorsement by HC and LTCF staff and examined the relationship between such myths and preparedness to help in a suicidal crisis.

**Methods:**

A total of 735 HC and LTCF staff in Västra Götaland County, Sweden, completed an anonymous questionnaire that included the Attitudes Toward Suicide Questionnaire. Data were collected between November 2023 and September 2025. Preparedness to help was defined as “I am prepared to help a person in a suicidal crisis by making contact and talking with him/her”. Logistic regression was used to examine associations between suicide myth endorsement and preparedness to help.

**Results:**

About one-third of participants endorsed myths such as suicide attempts are impulsive, threats are rarely acted upon, and suicide occurs without warning. Although participants’ answers showed that they were unsure about several myths, four in five felt prepared to help someone in a suicidal crisis. Greater myth endorsement was significantly associated with two-fold lower odds of being prepared to help, even after adjusting for demographic factors and professional background.

**Conclusions:**

Suicide myths are not uncommon among HC and LTCF staff and are linked to lower self-reported preparedness to help in suicidal crises. With one in five staff members feeling unprepared, our findings highlight the need for targeted staff training in suicide prevention within care settings for older adults.

**Supplementary Information:**

The online version contains supplementary material available at 10.1186/s12877-026-07393-y.

## Background

Suicide rates are the highest among women and men aged 65 and older across all regions of the world [1]. In Sweden, as in many other countries, suicide is recognized as a major public health issue, with men aged 85 and above showing the highest rates per capita in 2023 [2]. Older adults often require home care (HC) services or reside in long-term care facilities (LTCFs; [3], [4]). In 2023, 14% of Swedish residents aged 80 and above lived in LTCFs and an additional 18% received HC [5]. Institutional residency is associated with a fourfold increase in suicidal ideation among older adults [6]. A Swedish psychological autopsy study showed that half of those aged 75 or older who died by suicide received HC, compared to less than a third of those in the age- and sex-matched population-based comparison group [7]. A register-based study found that half of all suicides among older adults living in LTCFs occurred within a year of moving into the facility [8]. Given their close and continuous contact with service users, staff in HC and LTCFs are in a unique position to recognize early warning signs and contribute to suicide prevention efforts. Older adults are often referred to or admitted to HC and LTCF settings due to chronic illness, physical injury, functional decline, or cognitive impairment, all of which are established risk factors for suicide in later life [9]. Despite this elevated risk, routine suicide risk screening is not consistently implemented during referral or admission to HC and LTCF settings. As a result, the responsibility for identifying emerging suicidality often falls to frontline staff in the course of daily care. In residential settings, family members, if available, and fellow residents may only recognize signs of imminent danger, further underscoring the critical role of staff in early detection and prevention. However, their ability to fulfil this role may be hindered by limited knowledge, misconceptions, stigma, and insufficient organizational resources [10, 11]. A growing body of research has examined healthcare professionals’ attitudes towards self-harm and suicidal behaviour, demonstrating that such attitudes can influence clinical decision-making, empathy, and willingness to engage with suicidal individuals. Reviews indicate that negative attitudes or discomfort towards suicidal patients may adversely affect the therapeutic relationship and reduce confidence in intervention [12–14]. Conversely, greater knowledge about suicide and stronger belief in its preventability have been associated with higher perceived preparedness to help and more supportive responses to individuals expressing suicidal ideation [13, 14]. While much of this research has focused on hospital and psychiatric settings, considerably less attention has been directed towards staff working in HC and LTCFs, despite their frequent contact with older adults at elevated suicide risk. Emerging evidence from care home settings suggests that suicidal ideation in older adults may sometimes be normalized, reducing the perceived urgency to intervene and illustrating how underlying beliefs and assumptions about suicide can shape responses to individuals in crisis [15].

### Suicide myths and preparedness to help

One important dimension of healthcare staff attitudes towards suicide concerns endorsement of suicide myths and perceived preparedness to intervene. The World Health Organization [16] identified several common myths about suicide, including: “People who talk about suicide do not mean to do it”, “Most suicides happen suddenly without warning”, “Once someone is suicidal, he or she will always remain suicidal”, “Only people with mental disorders are suicidal” and “Talking about suicide is a bad idea and can be interpreted as encouragement”. While these myths may contain elements of truth in some cases, they generally misrepresent the nature of suicidality and can hinder proper detection and intervention. Dispelling such misconceptions is therefore essential for effective suicide prevention [17]. For example, research shows that suicidal communication is often a sign of psychological pain [18], and most suicides are preceded by identifiable warning signs [16]. Recent studies also indicate that debunking suicide myths may foster helping behaviours. In a nationally representative survey of Australian adults, debunking the myth that “asking about suicide might trigger suicidal thoughts” was most strongly associated with increased intentions to help [19]. Similarly, exposure to myth-debunking content improved suicide-related knowledge and intentions to help in a German adult sample [20]. Data from a national telephone survey showed greater willingness to help among individuals who held fewer misconceptions and stronger belief in suicide preventability [21]. In the context of LTCFs, two studies evaluated knowledge and attitudes about suicide myths before and after gatekeeper training sessions, both reporting significant improvements in post test scores compared to pretest scores [22, 23]. However, qualitative research in nursing home settings suggests that formal suicide prevention training remains limited, and that scepticism regarding the preventability of suicide may influence staff engagement with prevention initiatives [24].

While suicide myth debunking has become a widely used prevention strategy globally, and associations between myth endorsement and helping intentions have been demonstrated in general adult samples [19–21], the relationship between suicide myth endorsement and preparedness to intervene among HC and LTCF staff remains underexplored. The current study therefore sought to address this knowledge gap by focusing on a population that may serve as a key resource in suicide prevention among older adults. Specifically, it aimed to (a) assess the extent of agreement with suicide myths and the level of preparedness to help in a suicidal crisis among HC and LTCF staff, and (b) examine whether stronger agreement with suicide myths is associated with lower preparedness to help in a suicidal crisis, while controlling for age, gender, education level, work experience, and participation in short mental health/suicide training.

## Methods

### Study participants and procedure

Unit heads of HC services and LTCFs in Västra Götaland County, Sweden, were contacted by one of the researchers (SW) to participate in a study on attitudes towards mental illness and suicide. Information about the study was communicated either by telephone or by mail. Within participating facilities, questionnaires were distributed by unit heads during staff meetings, and those who wished to participate responded to the anonymous questionnaires individually during the meeting. Although exact attendance records were not available, unit heads estimated that approximately 25% of staff remained on the unit to maintain care while 75% attended the routine staff meeting and completed the questionnaires. These were returned to the unit head during the meeting and collected by one of the researchers (SW) who subsequently visited the facility. Data were collected between November 2023 and September 2025. A total of 793 individuals working in HC/LTCF participated in the study. Of these, 27 participants were excluded from the analysis due to missing data: 10 individuals because they responded with “Do not want to answer” only, 7 individuals provided no demographic information, and 10 individuals completed less than 10% of the survey. While unit heads of care were also invited to participate and 31 completed the questionnaire, this paper focuses solely on staff responses, providing a total of 735 questionnaires for analysis.

### Measures

#### Demographic variables

Gender options available were man, woman, and non-binary/other. Age group options were: 18–29, 30–44 and 45 + years. Level of education consisted of five options ranging from no formal education to university degree. Years of work experience were measured by the total number of years participants had worked in HC/LTCF.

#### Attitudes towards suicide

To measure attitudes towards suicide, we used the original Swedish version of the Attitudes Toward Suicide Questionnaire (ATTS; [25]). The ATTS consists of 37 items addressing different attitudes towards suicide as well as myths associated with suicide. Items are scored on a 5-point Likert scale (“agree completely”, “agree to a large extent”, “undecided”, “do not agree”, and “agree not at all”). In the current study, we used seven items from the ATTS to examine agreement with suicide myths:

- Item 8 “Most suicide attempts are impulsive actions”,
- Item 10 “Once a person has made up his/her mind about committing suicide no one can stop him/her”.
- Item 15 “There is a risk of evoking suicidal thoughts in a person’s mind if you ask about it”.
- Item 16 “People who make suicidal threats seldom complete suicide”.
- Item 25 “Once they have suicidal thoughts, a person will never let them go”.
- Item 26 “Suicide happens without warning”.
- Item 37 “People who talk about suicide do not commit suicide”.

To assess preparedness to help in a suicidal crisis, we used item 34 from the ATTS: “I am prepared to help a person in a suicidal crisis by making contact and talking with him/her.” Given the sensitivity of questions related to suicide, we also added the response option “Do not want to answer” to all items.

### Statistical analysis

First, prevalence rates of agreement with suicide myths and preparedness to help were calculated. Second, a mean score was created based on the seven ATTS items measuring attitudes towards suicide myths. Responses to item 34 “I am prepared to help a person in a suicidal crisis by making contact and talking with him/her” were dichotomized: participants who strongly agreed or agreed were categorized as more prepared to help, while those who were undecided, disagreed, or strongly disagreed were categorized as less prepared to help. Logistic regression was then used to estimate associations between agreement with suicide myth mean score and preparedness to help. Results are presented as odds ratios (ORs), i.e., representing the change in the odds of being less prepared to help associated with a one-unit increase in suicide myth endorsement. In the adjusted analysis, we entered age and gender as covariates into the model. In the fully adjusted analysis, we entered age, gender, education level, work experience, and previous participation in a short mental health/suicide training into the model. A two-tailed alpha level of 0.05 was used to determine statistical significance. Missing answers were excluded from all analyses. No imputation procedures were used. All statistical analyses were conducted using IBM SPSS Statistics version 30.0 [26].

## Results

### Demographic characteristics

Table 1 shows demographic characteristics of the study participants. The majority (87%) were women. The largest age group was 45 years and above, and almost three-quarters (72%) indicated Sweden as their nation of origin. Levels of education varied widely, from no formal education to having a university degree. In all, 35% of study participants worked in HC, 62% in LTCF, and 3% in both. Most participants (88%) were nursing assistants, out of which 10% had additional certifications or advanced training qualifications. Participants had an average of 14.5 years of work experience (*SD* = 11.7). 34% had previously undergone some form of brief training in mental health and suicide.

**Table 1:** Demographic characteristics of participants working in home care or long-term care facilities

Characteristics	*n*	%	Total	Missing	Do not want to answer
Gender			707	11	17
Women	614	86.8			
Men	91	12.9			
Non-binary/Other	2	0.3			
Age group			702	14	19
18–29	136	19.4			
30–44	229	32.6			
45+	337	48.0			
Nation of origin			686	12	37
Sweden	496	72.3			
Other Nordic countries	19	2.8			
Rest of the world	171	24.9			
Level of education			700	22	13
No formal education	3	0.4			
Compulsory school	30	4.3			
Upper secondary school	429	61.3			
Higher vocational education	162	23.1			
University	76	10.9			
Work setting			712	11	12
Home care	252	35.4			
Long term care facility	440	61.8			
Both	20	2.8			
Occupation			697	15	20
Care assistant	78	11.2			
Nursing assistant	615	88.2			
Nurse	4	0.6			

Valid proportions. Missing data excluded

*N = 735*

### Agreement with suicide myths and preparedness to help

Table 2 shows the prevalence of agreement with suicide myths and preparedness to help. The suicide myths with the highest levels of participant agreement were item 26 (“Suicide happens without warning.”; 36%), item 16 (“People who make suicidal threats seldom complete suicide.”; 32%), and item 8 (“Most suicide attempts are impulsive actions”; 27%). The myths with the highest level of disagreement were item 10 (“Once a person has made up his/her mind about committing suicide, no one can stop him/her.”; 44%), and item 15 (“There is a risk of evoking suicidal thoughts in a person’s mind if you ask about it.”; 41%). The “Undecided” responses accounted for between 34% and 48% of answers, depending on the item. Most respondents (80%) reported feeling prepared to help in a suicidal crisis, as reflected by answering “strongly agree/agree”.

**Table 2:** Agreement with suicide-related myths and preparedness to help measured by the Attitudes Toward Suicide Questionnaire

Items	Strongly agree/Agree (valid *n* %*)	Undecided (valid *n* %*)	Strongly disagree/Disagree (valid *n* %*)	*n*	Missing	Do not want to answer
Suicide myths						
8. Most suicide attempts are impulsive actions.	186 (27.2)	271 (39.6)	228 (33.3)	685	12 (1.6%)	38 (5.2%)
10. Once a person has made up his/her mind about committing suicide, no one can stop him/her.	156 (22.0)	244 (34.4)	309 (43.6)	709	4 (0.5%)	22 (3.0%)
15. There is a risk of evoking suicidal thoughts in a person’s mind if you ask about it.	149 (21.8)	255 (37.3)	280 (40.9)	684	4 (0.5%)	47 (6.4%)
16. People who make suicidal threats seldom complete suicide.	210 (31.5)	325 (44.2)	131 (19.7)	666	9 (1.2%)	60 (8.2%)
25. A person once they have suicidal thoughts will never let them go.	125 (18.4)	294 (43.3)	260 (38.3)	679	8 (1.1%)	48 (6.5%)
26. Suicide happens without warning.	239 (35.5)	229 (34.0)	205 (30.5)	673	16 (2.2%)	46 (6.3%)
37. People who talk about suicide do not commit suicide.	93 (13.9)	319 (47.6)	258 (38.5)	670	16 (2.2%)	49 (6.7%)
Preparedness to help						
34. I am prepared to help a person in a suicidal crisis by making contact and talking with him/her.	548 (80.4)	101 (14.8)	33 (4.8)	682	14 (1.9%)	39 (5.3%)

Missing values were excluded

* Valid percentages

Logistic regression showed that higher suicide myth endorsement was significantly associated with decreased preparedness to help someone in a suicidal crisis (Table 3). Specifically, each one-unit increase in the suicide myth endorsement mean score was associated with approximately twice the odds of being less prepared to help (OR = 2.03, 95% CI = [1.47, 2.8], *p* < .001). Adjustment for covariates (age, gender, education level, work experience, and previous participation in a short mental health/suicide training) did not substantially alter the association between suicide myths endorsement and preparedness to help, with confidence intervals remaining stable across models. To examine whether associations were sensitive to the categorization of the outcome variable, a sensitivity analysis was conducted treating “undecided” responses as a separate category. Higher suicide myth endorsement remained significantly associated with lower preparedness to help; each one-unit increase in the suicide myth mean score was associated with greater odds of being undecided rather than agreeing that one felt prepared to help (unadjusted OR = 2.32, 95% CI = [1.61, 3.35], *p* < .001), indicating that the findings were robust to alternative outcome categorizations. Unadjusted, partially adjusted, and fully adjusted models can be found in Supplementary Table 1.

**Table 3:** Association between agreement towards suicide myths and preparedness to help in a suicidal crisis

Predictor	Unadjusted OR (95% CI)	Adjusted OR* (95% CI)	Fully adjusted OR† (95% CI)
Suicide myths mean score	2.03 (1.47–2.80)	2.01 (1.44–2.79)	1.72 (1.17–2.51)

*OR *odds ratio, *CI *confidence interval

* Adjusted for age and gender

† Adjusted for age, gender, education level, work experience in years, and participation in mental health and suicide training

To further explore whether background characteristics were associated with suicide myth endorsement and preparedness to help, additional regression analyses were conducted (Supplementary Table 2). Years of work experience was the factor most consistently associated with suicide myths. A greater number of years of work experience was associated with lower agreement with the statement that asking about suicide may evoke suicidal thoughts in a person’s mind (OR = 0.95, 95% CI [0.92, 0.97], *p* < .05).

## Discussion

In this quantitative questionnaire study of attitudes towards suicide among 735 HC and LTCF staff, approximately one in three endorsed the myths that suicide occurs without warning, that suicidal threats are rarely followed by action, and that most suicide attempts are impulsive. Participants most strongly disagreed with the myths that individuals determined to die by suicide cannot be stopped, and that asking about suicide can trigger suicidal thoughts. Between one-third and one-half of participants remained unsure about some of the myth statements. Despite this uncertainty, four out of five reported feeling prepared to help someone in a suicidal crisis. Regression analyses further showed that greater endorsement of suicide myths was significantly associated with lower preparedness to help, even after adjusting for demographic factors and professional background.

A previous study [27] investigated attitudes towards suicide myths in the general population in Norway, a country similar to Sweden in many aspects. Using the same questionnaire with comparable items, they found generally lower levels of agreement with myths than those observed in our study. This finding is somewhat unexpected, given that healthcare professionals can be assumed to have greater awareness and knowledge about suicide than the general population. One possible explanation for this unexpected finding is that many individuals working in care settings receive little to no formal training in suicide prevention, as reflected by the fact that only one third of participants in our study reported having attended any form of brief mental health or suicide-related training. Staff in HC/LTCFs may also be more focused on somatic care, with less emphasis on mental health. Related, ageism may also have influenced the higher level of adherence to certain myths about suicide, i.e., believing that older adults who express suicidal thoughts are simply “ready to die” or that such thoughts are understandable and not preventable. This has been recently highlighted by research involving nursing home professionals, indicating that acceptance of suicidal ideation as a normal part of ageing may reduce the perceived urgency to intervene, thereby reinforcing age-based biases in suicide prevention practices within care homes [15]. Furthermore, stigma and discomfort surrounding discussions of suicide may persist even among healthcare professionals. For example, the authors of a recent review [12] highlight that some healthcare professionals may display limited empathy and negative attitudes towards patients exhibiting suicidal behaviours, which can adversely affect the professional-patient relationship. Differences in sample characteristics may also help explain the discrepancy between our study findings and those obtained in the general population in Norway [27]. For instance, two thirds of the participants in our sample had no vocational or university training beyond high school, and lower levels of educational attainment have been linked to lower mental health literacy [28]. Finally, psychological factors may also play a role. For example, some staff may unconsciously endorse suicide myths as a psychological defence mechanism to protect themselves from feelings of helplessness when caring for suicidal patients [29], particularly while working under the strained workload that characterizes these settings [30].

Notably, between one-third and one-half of participants were undecided about the suicide myth statements, particularly those suggesting that people who threaten or talk about suicide rarely die by suicide. These relatively high proportions of undecided responses may reflect limited knowledge and confidence in evaluating suicide-related statements, consistent with the overall lack of suicide prevention training among participants. However, in some cases, being undecided may also reflect a deeper understanding of the topic’s complexity. Some items such as “Most suicide attempts are impulsive actions” may have been perceived as ambiguous, leading respondents to adopt a neutral position. Lastly, answering “undecided” may also reflect response bias or a desire to appear neutral when unsure about the “correct” answer. In addition to staff being undecided on many of the suicide myths, 15% of respondents were also ambivalent about their preparedness to help someone in a suicidal crisis. In this case, neutral responses may not reflect a passive attitude towards suicide prevention, but rather a sense of perceived lack of control [31]. Perceived lack of control may be a consequence of several factors related to working within HC and LTCF, such as a lack of formal training and resources [10], as well as limited time for discussion and supervision [11]. Given that HC and LTCF staff represent the main source of social interaction for older adults using these services [32], staff should be made aware of their crucial role in suicide prevention [33]. Providing adequate training, support, and time to engage in difficult conversations with distressed individuals is therefore essential to strengthen suicide prevention efforts.

Despite the fact that four in five participants reported that they felt prepared to assist someone in a suicidal crisis, regression analyses indicated that greater endorsement of suicide myths was significantly associated with lower preparedness to help, even after controlling for age, gender, education level, work experience, and prior mental health and suicide prevention training. This finding aligns with previous general population-based research demonstrating that lower endorsement of suicide myths is associated with greater preparedness to support someone in a suicidal crisis [19–21]. Our results support and extend this body of literature by showing that, while self-perceived preparedness is generally high, misconceptions about suicide may undermine actual readiness or confidence to intervene effectively, even among HC and LTCF staff who might be expected to have greater awareness of these topics. These findings underscore the need for rigorous evaluation of existing suicide prevention training targeting staff who work with older adults in care settings [33]. Importantly, the association between suicide myth endorsement and preparedness to help remained robust in sensitivity analyses in which “undecided” responses were treated as a separate outcome category. Higher suicide myth endorsement was similarly associated with greater odds of being undecided rather than agreeing to feeling prepared to help. This suggests that suicide myths are linked not only to explicitly lower preparedness, but also to uncertainty regarding one’s ability to intervene, further reinforcing the potential impact of misconceptions on suicide prevention efforts within HC and LTCF settings.

Consistent with our results, previous qualitative research found that only one third of interviewed caregivers in nursing homes had received mental health and/or suicide prevention training [24]. In that study, resistance to participation often stemmed from doubts about the preventability of suicide, while some professionals expressed a desire for further education in suicide prevention. Future research should therefore not only evaluate the effectiveness of existing training programs but also explore the psychological and organizational mechanisms that sustain suicide myths and shape staff attitudes towards suicide prevention within care settings. Moreover, structural barriers to suicide-preventative work, such as limited time and workload pressures, should also be investigated. Time for dialogue has been identified as a key resource in suicide prevention, along with staff capacity for action and work experience [11]. In that study, participants reported that greater work experience facilitated addressing suicidal ideation and depressive symptoms among service users. In our supplementary analyses, greater work experience was likewise associated with lower endorsement of several suicide myths, including the belief that asking about suicide may provoke suicidal thoughts. This suggests that accumulated clinical experience may contribute to a more accurate understanding of suicide and reduce endorsement of common misconceptions. However, work experience was not independently associated with preparedness to help in these additional analyses, indicating that experience alone may not be sufficient to enhance self-perceived readiness to intervene. Taken together, these findings suggest that even experienced staff may benefit from additional structured support and training to strengthen suicide prevention efforts in HC and LTCF settings.

### Methodological considerations

Strengths of the current study include its relatively large sample size and focus on healthcare staff working with older adults in HC and LTCFs. Although the sample was not randomly selected, it included staff from a wide range of workplaces throughout the region, offering good cross-section of those working in HC and LTCFs, thereby enhancing the generalizability of the findings. Notably, few previous studies in this field have included such a large sample. For example, one similar survey-based study [30] had about 150 participants, making the present study relatively unique in its scope among questionnaire-based research in Swedish care for older adults. Nevertheless, several limitations should be noted. Preparedness to help was assessed using a single item focused solely on “talking” to someone in a suicidal crisis (“I am prepared to help a person in a suicidal crisis by making contact and talking with him/her.”). This excludes other important forms of support, such as listening, observing, or referring an individual to professional services. Although we argue that talking is central to most supportive interactions, a more comprehensive measure of preparedness could be developed for future studies. In addition, the term “suicidal crisis” was not explicitly defined, which may have resulted in varied interpretations among participants. For example, if understood as an ongoing suicide attempt, participants may have perceived talking as an inadequate response, whereas if interpreted as the expression of suicidal thoughts, talking may have seemed more appropriate. All self-report items in this study are also potentially subject to social desirability bias. Although questionnaires were filled out during staff meetings and participation was anonymous, some respondents may have felt obliged to participate or feared that their responses could be traced back to them. Related, cultural factors may also have influenced responses, as approximately one quarter of participants were born outside Scandinavia, where suicide may be a more sensitive or stigmatized topic. It is therefore possible that individuals who chose the undecided responses differed systematically from those who did. For example, staff members holding strong moral, religious, or personal reservations about discussing suicide may have been more inclined to choose the “undecided” response. Finally, the Attitudes Toward Suicide Questionnaire assesses general beliefs about suicide myths and preparedness to help, rather than factors specific to healthcare staff. While it does not explicitly focus on suicidal crises in older adults, it is possible that respondents considered this population when completing the questionnaire. Future research may benefit from developing instruments tailored to the context of older adult care to better capture domain-specific attitudes and competencies.

## Conclusions

Findings highlight the persistence of suicide myths among staff working in HC and LTCFs and their potential impact on perceived preparedness to help individuals in suicidal crisis, suggesting that such misconceptions may obstruct suicide preventative efforts in HC and LTCF settings. Therefore, addressing suicide myths among staff should be viewed as a key component within a broader, multifaceted approach to improving mental health and reducing suicidality among older adults. Future work should focus on identifying effective strategies to dispel these myths among staff, for example by evaluating the impact of different educational formats, such as workshops or discussion-based training in order to improve knowledge, attitudes, and intervention confidence.

## Supplementary Information

Supplementary Material 1.

## Data Availability

The data that support the findings of this study are available from the corresponding author but restrictions apply to the availability of these data, which were used under license for the current study, and so are not publicly available. Data are however available from the authors upon reasonable request and with permission of the Swedish National Ethics Review Board.

## Abbreviations

ATTS: Attitudes Toward Suicide Questionnaire
CI: Confidence interval
HC: Home care
LTCF: Lome-term care facility
OR: Odds ratio
