# Supplementary material for: Suicide myths and preparedness to help among staff at home care and long-term care facilities in Sweden: a quantitative questionnaire-based study
Source: BMC Geriatr. 2026 Apr 2;26:490. doi: 10.1186/s12877-026-07393-y (PMC13064347; doi:10.1186/s12877-026-07393-y)
Supplement: Supplementary file 1 — Supplementary Material 1. [file 12877_2026_7393_MOESM1_ESM.docx]

| **Supplementary Table 1.** Association between agreement towards suicide myths and being undecided rather than agreeing to be prepared to help in a suicidal crisis | | | |
| --- | --- | --- | --- |
| **Predictor** | **Unadjusted OR (95% CI)** | **Adjusted OR***  **(95% CI)** | **Fully adjusted OR†**  **(95% CI)** |
| Suicide myths mean score | 2.32 (1.61 – 3.35) | 2.33 (1.60 – 3.38) | 1.94 (1.25 – 3.00) |
| *Note.* OR=odds ratio. CI=confidence interval.  * Adjusted for age and gender  † Adjusted for age, gender, education level, work experience in years, and participation in mental health and suicide training | | | |

| **Supplementary Table 2.** Associations between background factors and agreement towards suicide myth items and preparedness to help in a suicidal crisis | | | | | | | | |
| --- | --- | --- | --- | --- | --- | --- | --- | --- |
| **Background variable*** | Item 8  OR (95% CI) | Item 10  OR (95% CI) | Item 15  OR (95% CI) | Item 16  OR (95% CI) | Item 25  OR (95% CI) | Item 26  OR (95% CI) | Item 37  OR (95% CI) | Item 34**  OR (95% CI) |
| **Age category** |  |  |  |  |  |  |  |  |
| Age 18–29† | 1 | 1 | 1 | 1 | 1 | 1 | 1 | 1 |
| Age 30–44 | 1.24 (0.71–2.19) | 0.98 (0.54–1.78) | 1.29 (0.73–2.28) | 1.14 (0.66–1.98) | 1.40 (0.73–2.68) | 0.73 (0.43–1.24) | 1.02 (0.46–2.27) | 1.19 (0.59–2.39) |
| Age 45+ | 1.54 (0.82–2.89) | 1.35 (0.70–2.62) | 1.00 (0.52–1.93) | 0.92 (0.49–1.71) | 1.49 (0.73–3.04) | 0.84 (0.47–1.51) | 1.62 (0.70–3.75) | 1.62 (0.76–3.44) |
| **Gender** |  |  |  |  |  |  |  |  |
| Male† | 1 | 1 | 1 | 1 | 1 | 1 | 1 | 1 |
| Female | 0.90 (0.50–1.62) | 1.01 (0.53–1.91) | 1.22 (0.64–2.31) | 0.60 (0.34–1.06) | 1.91 (0.86–4.25) | 1.56 (0.85–2.87) | 0.36 (0.14–0.95) | 0.93 (0.45–1.90) |
| **Education level** |  |  |  |  |  |  |  |  |
| No formal education or compulsory school† | 1 | 1 | 1 | 1 | 1 | 1 | 1 | 1 |
| Upper secondary school | 0.58 (0.22–1.56) | 0.80 (0.30–2.13) | 0.78 (0.27–2.28) | 2.07 (0.66–6.47) | 0.49 (0.18–1.33) | 0.71 (0.30–1.68) | **0.36 (0.14–0.95)** | 0.86 (0.28–2.68) |
| Higher vocational education or university | 0.57 (0.21–1.55) | 0.58 (0.21–1.61) | 0.71 (0.24–2.11) | 1.84 (0.57–5.92) | 0.47 (0.17–1.32) | 0.54 (0.22–1.32) | **0.31 (0.11–0.85)** | 0.93 (0.29–2.97) |
| **Work experience in years** | **0.98 (0.96–0.998)** | 0.99 (0.97–1.01) | **0.95 (0.92–0.97)** | 1.02 (0.997–1.04) | **0.97 (0.95–0.996)** | 1.01 (0.99–1.03) | 1.00 (0.98–1.03) | 1.00 (0.97–1.02) |
| **Participation in mental health and suicide training** | **1.54 (1.04–2.28)** | 1.15 (0.75–1.75) | 0.93 (0.59–0.59) | 0.80 (0.54–1.18) | 0.84 (0.52–1.35) | 0.93 (0.64–1.35) | 1.07 (0.64–1.81) | 0.87 (0.54–1.40) |
| *Note.* OR=odds ratio. CI=confidence interval. Bold estimates indicate significance at the 0.05 level.  † Reference category  * Item 8 “Most suicide attempts are impulsive actions”  Item 10 “Once a person has made up his/her mind about committing suicide no one can stop him/her”  Item 15 “There is a risk of evoking suicidal thoughts in a person’s mind if you ask about it”  Item 16 “People who make suicidal threats seldom complete suicide”  Item 25 “Once they have suicidal thoughts, a person will never let them go”  Item 26 “Suicide happens without warning”  Item 37 “People who talk about suicide do not commit suicide”  ** Item 34 “I am prepared to help a person in a suicidal crisis by making contact and talking with him/her.” This item was reverse coded for analysis; thus, odds ratios reflect the likelihood of being less prepared to help. | | | | | | | | |
